# Innovative Solid Lipid Nanoparticle-Enriched Hydrogels for Enhanced Topical Delivery of L-Glutathione: A Novel Approach to Anti-Ageing

**DOI:** 10.3390/pharmaceutics17010004

**Published:** 2024-12-24

**Authors:** Mengyang Liu, Manisha Sharma, Guoliang Lu, Zhiwen Zhang, Wenting Song, Jingyuan Wen

**Affiliations:** 1 School of Pharmacy, Faculty of Medical and Health Sciences, The University of Auckland, Auckland 1023, New Zealand; m.liu@auckland.ac.nz (M.L.); manisha.sharma@auckland.ac.nz (M.S.); 2 Auckland Cancer Society Research Centre, Faculty of Medical and Health Sciences, University of Auckland, Auckland 1023, New Zealand; gl.lu@auckland.ac.nz; 3 Maurice Wilkins Centre for Molecular Biodiscovery, The University of Auckland, Auckland 1023, New Zealand; 4 Department of Pharmaceutics, School of Pharmacy, Fudan University, Shanghai 200433, China; zhangzhiwen@fudan.edu.cn; 5 Department of Pharmaceutics, School of Pharmacy, China Pharmaceutical University, Nanjing 211198, China

**Keywords:** glutathione, antioxidant, topical delivery, hydrogel, solid lipid nanoparticles, anti-ageing

## Abstract

**Background:** Skin ageing, driven predominantly by oxidative stress from reactive oxygen species (ROS) induced by environmental factors like ultraviolet A (UVA) radiation, accounts for approximately 80% of extrinsic skin damage. L-glutathione (GSH), a potent antioxidant, holds promise in combating UVA-induced oxidative stress. However, its instability and limited penetration through the stratum corneum hinder its topical application. This study introduces a novel solid lipid nanoparticle (SLN)-enriched hydrogel designed to enhance GSH stability, skin penetration, and sustained release for anti-ageing applications. **Methods:** GSH-loaded SLNs were prepared via a double-emulsion technique and optimized using factorial design. These SLNs were incorporated into 1–3% (*w*/*v*) Carbopol hydrogels to produce a semi-solid formulation. The hydrogel’s characteristics, including morphology, mechanical and rheological properties, drug release, stability, antioxidant activity, cytotoxicity, and skin penetration, were evaluated. **Results:** SEM and FTIR confirmed the uniform dispersion of SLNs within the hydrogel. The formulation exhibited desirable properties, including gel strength (5.1 ± 0.5 g), spreadability (33.6 ± 1.9 g·s), pseudoplasticity, and elasticity. In vitro studies revealed a biphasic GSH release profile, with sustained release over 72 h and over 70% cumulative release. The hydrogel significantly improved antioxidant capacity, protecting human fibroblasts from UVA-induced oxidative stress and enhancing cell viability. Stability studies indicated that 4 °C was optimal for storage over three months. Notably, the hydrogel enhanced GSH penetration through the stratum corneum by 3.7-fold. **Conclusions:** This SLN-enriched hydrogel effectively improves GSH topical delivery and antioxidant efficacy, providing a promising platform for anti-ageing and other bioactive compounds with similar delivery challenges.

## 1. Introduction

Skin ageing manifests through various noticeable and measurable changes, including loss of elasticity, thinning of skin tissue, compromised barrier function, uneven keratinization, darker skin tone, and reduced lipid content [1,2,3,4]. This complex process is influenced by genetic and environmental factors, primarily the cumulative damage caused by ultraviolet A (UVA) radiation exposure [5,6]. To mitigate skin ageing, antioxidants have been extensively employed by the cosmetic and pharmaceutical industries worldwide.

L-glutathione (GSH), widely referred to as the “mother of all antioxidants,” protects cells by scavenging free radicals, such as superoxide (O2−•) and hydroxyl (•OH), and detoxifies organs, like the kidney, liver, lung, and intestine, commonly exposed to xenobiotics [7,8,9,10,11]. A decline in GSH levels is associated with various degenerative diseases and skin disorders [12,13,14]. However, GSH’s instability and poor penetration through the skin’s stratum corneum (SC) pose significant challenges for its topical application [10,15,16,17,18,19,20,21]. A lipid-based nanoscale carrier system, such as solid lipid nanoparticles (SLNs), is a promising solution for enhancing the stability and delivery of GSH due to the lipophilic nature of the skin barrier [10,11]. Chitosan-coated SLNs, with sizes ranging from 50 to 1000 nm, offer advantages such as improved physical stability, lower toxicity, enhanced protection of active compounds, and ease of scaling up [3,22]. Chitosan, a cationic natural biopolymer derived from chitin, enhances drug permeability and provides positive zeta potential to prevent particle aggregation alongside its biocompatibility, stability, and non-toxic profile [23]. Despite their advantages, SLNs in dispersion form have low viscosity, limiting their direct applicability to the skin surface. Incorporating SLNs into a semi-solid matrix like hydrogels addresses this limitation [24,25]. Hydrogels, characterized by their cross-linked polymeric structure, provide numerous benefits for topical delivery, including enhanced retention, sustained drug release, and ease of application. Their soft texture and high water content contribute to improved patient compliance and reduced irritation [26,27,28]. When SLNs are dispersed in hydrogels, the combined system leverages the strengths of both carriers, addressing limitations such as the low viscosity and poor adhesion of SLN dispersions [29].

Polymers like Carbopol play a crucial role in forming hydrogel matrices. Carbopol, an anionic polymer, imparts desirable rheological properties, ease of preparation, and excellent swelling capacity, making it an ideal candidate for semi-solid formulations [30,31]. Hydrogels, known for their versatile applications, typically exhibit mesh sizes of up to 100 nm, as reported in the literature [32]. The mesh size of hydrogels depends on their degree of swelling; greater swelling correlates with larger mesh sizes [33,34]. Consequently, the secondary release mechanism of hydrogels is governed by swelling dynamics. Three critical factors influence the rate and extent of swelling: (1) the degree of cross-linking; (2) the chemical structure of the polymer; and (3) external conditions, such as temperature, pH, humidity, and ionic strength [35,36]. In addition to swelling, chemical-based controlled-release mechanisms contribute to drug delivery. These mechanisms involve the enzymatic breakdown of polymeric chains, releasing active compounds encapsulated within the hydrogel’s network structure. The advantages of employing hydrogel systems for topical delivery are numerous and well-documented: (1) Enhanced retention time: Hydrogels improve the retention time of solid lipid nanoparticle (SLN) carrier systems at the application site, optimizing therapeutic efficacy [37]; (2) Sustained drug release: They facilitate controlled and prolonged release, ensuring consistent drug delivery over time [38]; (3) Reduced side effects: Compared to traditional dosage forms, hydrogels are associated with fewer adverse effects, enhancing patient safety [39]; (4) Improved patient compliance: Their ease of application and reduced frequency of administration contribute to better adherence to treatment regimens [40]; (5) Ease of preparation and scalability: Hydrogels are straightforward to manufacture and scale up for commercial production, making them a practical choice for pharmaceutical applications [41]; (6) Cost-effectiveness: The materials required for hydrogel preparation are relatively inexpensive, contributing to their economic viability [42]; (7) Enhanced drug permeability: Certain gelling polymers within hydrogels can improve the permeability of drugs, facilitating better absorption and bioavailability [43]; (8) Comfortable application: The high water content of hydrogels creates a soothing and comfortable sensation on the skin, enhancing user satisfaction [44]; (9) Improved adhesion and bioadhesion: Hydrogels exhibit strong adhesive and bioadhesive properties, ensuring effective localization at the target site [45,46].

In this follow-up study, we build on our previous work optimizing L-glutathione-loaded solid lipid nanoparticles (SLNs) to develop a more advanced delivery system by integrating these SLNs into hydrogels for enhanced topical application. Our earlier study demonstrated that SLNs effectively improved the stability and encapsulation efficiency of GSH, with chitosan-modified SLNs offering superior antioxidant activity and compatibility with the skin barrier. This research advances those findings by incorporating optimized chitosan-coated SLNs into Carbopol-based hydrogels, overcoming the challenges of low viscosity and adhesion of SLN dispersions. The combined system leverages SLNs’ protective and permeation-enhancing properties with the retention, spreadability, and controlled-release advantages of hydrogels. We characterized the resulting GSH-SLN-enriched hydrogel (GSH-SLN-EH) for physicochemical stability, rheological properties, antioxidant efficacy, and skin penetration, aiming to enhance GSH delivery to combat oxidative-stress-induced skin ageing.

## 2. Materials and Methods

### 2.1. Materials

Reduced L-glutathione (GSH), stearic acid, and surfactants, including sorbitane trioleate (Span^®^ 85), polyoxyethylene sorbitan monooleate (Tween^®^ 80), chitosan, and cholesterol, were purchased from Sigma-Aldrich (Sigma, San Jose, CA, USA). Syringe filters (0.45 μm) were obtained from Membrane Solutions (Membrane Solutions, Seattle, WA, USA). Carbopol^®^ 971P NF polymer was supplied by Lubrizol (Wickliffe, OH, USA). Trifluoroacetic acid (TFA) and trichloroacetic acid (TCA) were purchased from Fluka (Honeywell Fluka, Seelze, Germany). Methanol, acetonitrile (ACN), and 3-(4,5-dimethylthiazol-2-yl)-2,5-diphenyltetrazolium bromide (MTT) were purchased from Merck (Merck, Darmstadt, Germany). Cell culture chemicals, including Phosphate-Buffered Saline (PBS), Dulbecco’s Modified Eagle Medium (DMEM), trypsin–EDTA, Fetal Bovine Serum (FBS), Non-essential Amino Acid solution (NEAA), and penicillin–streptomycin, were all purchased from Invitrogen (Invitrogen, Auckland, New Zealand). All other reagents and chemicals were of analytical grade.

### 2.2. Analytical Method of GSH

The RP-HPLC analytical method for GSH was developed in a prior study [16]. In brief, the HPLC system consisted of an Agilent HP 1200 series separation module (Agilent Corporation, Waldbronn, Germany) coupled with an Agilent vacuum degasser, quaternary pump, auto-sampler, thermos-stated column compartment, and photodiode array (PDA) detector, with data acquisition by Chemstation software (version 4.03). Analysis was performed by a reverse-phase HPLC assay using a Gemini C18 column (250 × 4.60 mm, 5 µm; Phenomenex, Torrance, CA, USA) fitted with a C18 guard column (10 × 3.0 mm; Phenomenex, Torrance, CA, USA), maintained at 25 ± 1 °C. Ultraviolet detection was performed at 215 nm. Mobile phase composition was 20:80 (*v*/*v*), 0.085% (*v*/*v*) TFA in ACN: 0.100% (*v*/*v*) TFA in water at a flow rate of 0.5 mL/min with 20 mL injection volume.

### 2.3. Preparation of GSH-Loaded Solid Lipid Nanoparticle-Enriched Hydrogels (GSH-SLN-EH)

#### 2.3.1. Fabrication of Hydrogels

Carbopol^®^ 971 was selected as the polymer for hydrogel preparation. The hydrogels were formulated at room temperature using three different concentrations of Carbopol^®^ 971 (1%, 3%, and 5% *w*/*v*). The polymer was gradually added to a mixture of phosphate-buffered saline (PBS, pH 5.5) and propylene glycol in a 4:1 (*w*/*w*) ratio. The resulting mixture was stirred continuously for 8–10 h using a magnetic stirrer to ensure homogeneity. Prior to application on the skin, the hydrogel pH was adjusted to a range of 5.0–5.5, suitable for human skin, by incorporating an appropriate amount of acidic (HCl) or basic (NaOH) solution. The prepared hydrogels were then stored at room temperature for 12 h to allow for swelling and equilibration, ensuring a stable and consistent formulation [47].

#### 2.3.2. Fabrication of GSH-SLN-EH

GSH-SLNs were prepared following a previously established protocol [22,48]. Briefly, 0.6 g of stearic acid was heated above its melting point (71 °C) and blended with 10% or 5% (*v*/*w*) of Span^®^ 85, a surfactant with a low hydrophilic–lipophilic balance (HLB) value. The molten mixture was stirred using a magnetic stirrer, and an aqueous solution (0.2 mL) containing 20 or 40 mg/mL of reduced L-glutathione (GSH) was gradually added. This mixture was subjected to ultrasonication (Fischer Scientific, Sonic Dismembrator Model 500, Hampton, NH, USA) at 45% or 55% amplitude for 1–2 min, forming the primary emulsion (W1/O). The primary emulsion was subsequently combined with 6.0 mL of a preheated (50 °C) 10% (*v*/*v*) Tween^®^ 80 Milli-Q water solution containing 0.1% or 0.2% (*w*/*v*) chitosan. Ultrasonication was applied at the same amplitude using a pulse program (10 s on, 5 s off) for 2–5 min, creating the double emulsion (W1/O/W2). This double emulsion was poured into 90 mL of cold water (4 °C) and stirred overnight to solidify the SLNs [49]. The GSH-SLN dispersion was frozen at −80 °C overnight and subsequently freeze-dried using a Labconco freeze dryer (Kansas City, MO, USA) at −60 °C and 0.233 mbar for 12 h. The resulting SLN powder was analyzed for moisture content using thermogravimetric analysis (TGA, Perkin Elmer 8000, Waltham, MA, USA). Finally, the dried GSH-SLN powder was incorporated into the hydrogel matrix by mixing under magnetic stirring at 600 rpm for 2 h, yielding the final GSH-SLN-enriched hydrogel (GSH-SLN-EH).

### 2.4. Characterisation Studies of GSH-Loaded Solid Lipid Nanoparticle-Enriched Hydrogels (GSH-SLN-EH)

#### 2.4.1. Morphological Study

The morphology of the GSH-SLN-enriched hydrogels (GSH-SLN-EH) was analyzed using scanning electron microscopy (SEM, Philips XL30S FEG, Eindhoven, the Netherlands). The hydrogel samples were freeze-dried (Labconco, Missouri, USA) at −60 °C and 0.233 mbar for 12 h until the moisture content was negligible (<5%), as verified through thermogravimetric analysis (TGA). Approximately 10 mg of the dried hydrogel was placed on aluminum stubs and sputter-coated with gold–palladium for SEM imaging.

#### 2.4.2. FTIR Analysis

Fourier-transform infrared (FTIR) spectroscopy was conducted to identify chemical interactions within the formulation. The analysis included samples of pure GSH, GSH-SLN, Carbopol^®^ 971, GSH-SLN-EH, and dried SLN-enriched hydrogels (SLN-EH), along with background scans. Pure GSH was analyzed in the wavenumber range of 4000 cm^−1^ to 500 cm^−1^. Measurements were performed at room temperature using OPUS software (version 6.5, Germany), with 25 scans recorded for each sample and the background.

#### 2.4.3. Texture Profile Analysis

The mechanical properties of GSH-SLN-EH, including gel strength (firmness), stickiness, adhesion, and spreadability, were evaluated using a texture analyzer (TA.XT, Stable Micro Systems, Surrey, UK). Three formulations containing 1%, 3%, and 5% Carbopol^®^ 971 were compared with a commercial gel control (Merino^®^ 97% Pure Aloe Vera Gel, Auckland, New Zealand).

##### Gel Strength

Gel strength was measured using a 10 mm Delrin cylinder probe in triplicate. A 10 mL sample of GSH-SLN-EH was placed in a glass vial on the texture analyzer platform. Gel strength was defined as the maximum force required for the probe to penetrate the hydrogel to a depth of 5 mm. The resultant force–distance plots were analyzed using Exponent^®^ 32 software to determine gel firmness.

##### Gel Spreadability

The spreadability of the three GSH-SLN-EH formulations (containing 1%, 3%, and 5% Carbopol^®^ 971) was evaluated alongside a commercial control gel (Merino^®^ 97% Pure Aloe Vera Gel, Auckland, New Zealand) using a TTC spreadability rig. The rig was equipped with attachments coded under the Heavy-Duty Platform (HDP) prefix and operated in conjunction with the 90 HDP configuration. Measurements were conducted in triplicate. For testing, 10 mL of GSH-SLN-EH was placed in a glass vial on the texture analyzer platform, ensuring contact with the probe. The spreadability was assessed based on the maximum work required for the probe to spread the hydrogel outwards from the HDP setup. The spreadability was calculated as the work performed during the probe’s interaction with the hydrogel, providing a quantifiable measure of its ease of application.

#### 2.4.4. Rheological Studies

##### Flow Behavior and Thixotropic Properties

Type of flow behavior and thixotropic properties of three GSH-SLN-EH and one control gel (Merino^®^ 97% Pure Aloe Vera Gel, Auckland, New Zealand) were conducted by Rheometric Scientific ARES and AR-G2 Discovery 2000 rheometers (TA Instruments, Melbourne, Australia) equipped with a Peltier plate temperature control (RTE 130 temperature controller) at 25 °C. Parallel steel plate geometry (40 mm diameter, TA Instruments, Melbourne, Australia) was used to carry out the flow sweep test. The flow sweep test was performed over the logarithmic shear rate that ranged from 1 to 400 1/s with reversed 400 to 1 1/s. In each case, the thixotropic properties of at least five replicates were examined. There are four different types of flow behaviors, namely in Bingham, Pseudoplastic, Newtonian, and Dilatant, respectively [50]. For Pseudoplastic fluids, thixotropic behaviors occur, which is defined as a time-dependent change in viscosity: ‘the longer the fluid undergoes shear stress, the lower its viscosity’ [51].

##### Viscoelasticity Properties

The viscoelasticity properties of three GSH-SLN-EH and a control gel (Merino^®^ 97% Pure Aloe Vera Gel, Auckland, New Zealand) were investigated by an AR2000 rheometer (TA Instruments, Melbourne, Australia) equipped with a Peltier plate temperature control (RTE 130 temperature controller, Melbourne, Australia) at 25 °C. Parallel steel plate geometry (40 mm diameter, TA Instruments, Melbourne, Australia) was used to carry out the oscillation frequency test. The oscillation test was performed after determination of its linear viscoelastic region, where stress was directly proportional to strain, and the storage modulus remained constant. A frequency sweep analysis was performed over the logarithmic frequency range from 0.1 to 100.0 rad/s angular frequency. Storage modulus (G’) and loss modulus (G”), the dynamic viscosity (ŋ*), and the phase angle (δ) were determined. In each case, the dynamic rheological properties of at least five replicates were examined. In brief, the parameters of storage or elastic modulus and loss or viscous modulus are derived from experiments with different phase angles, and therefore, the results are able to identify whether viscous or elastic behavior is dominant under any given condition [52]:

If G’ > G”, then elastic properties dominate, and the sample exhibits solid-like behavior.

If G’ < G”, then viscous properties dominate, and the sample exhibits liquid-like behavior.

#### 2.4.5. In Vitro Release Studies

A Franz cell (FDC-6, Logan instrument Corp, USA) was used in conducting in vitro drug release studies [5,22]. In brief, 2.5 mL GSH-SLN-EH (containing 2 mg of GSH according to the GSH-SLN-EH preparation) was placed into the donor chamber. The receptor chamber was filled with 12 mL of PBS (pH 5.5, skin condition). The donor temperature was set at 34 ± 0.5 °C using a water bath. The donor and receptor chambers were separated by a layer of synthetic cellulose membrane (14,000 MWCO, pre-soaked in PBS of pH 5.5 for 6 h). At predetermined time points (15 min, 30 min, and 1, 2, 3, 6, 9, 12, 24, and 48 h), aliquots (400 μL) were withdrawn from the receptor chambers and replaced with an equal volume of fresh PBS (pH 5.5) to maintain sink conditions. The aliquots were analyzed for drug concentration using an HPLC method as previously described. To investigate the mechanism behind the drug release profile of GSH-SLN-EH, these release results were modelled into various mathematical release models, such as the Korsmeyer–Peppas model and others [53]. Based on the comparison of modelling fit linear regression, the fit model was selected to illustrate the drug release mechanism.

#### 2.4.6. Stability Studies

A short-term accelerated stability test was conducted to evaluate the physicochemical stability of the optimized GSH-SLN-EH. In brief, GSH products were prepared into amber vials and stored in stability chambers at three different temperatures (4, 25, and 40 °C) according to ICH guidelines and British Pharmacopoeia (BP) [54,55]. Characterization parameters including particle size, zeta potential, drug content of SLNs, and gel strength, gel spreadability, and gel viscoelasticity were investigated at specified time intervals (0, 1, 2, and 3 months) in triplicates.

### 2.5. In Vitro Cell Culture Investigation

#### 2.5.1. Cell Culture

Skin fibroblast (Fbs) cells were cultured in DMEM medium, which is composed of 10% FBS, 1% non-essential amino acid (NEAA), and 1% penicillin–streptomycin (antibiotics) with pH values maintained at 7.4 ± 0.2. The cells were incubated in an incubator at 37 °C in the presence of 5% CO_2_. Fbs cells were washed with PBS and cultured with fresh cell medium every three days until the cell confluence reached 80% for further in vitro investigations.

#### 2.5.2. Cytotoxicity Study

The cytotoxicity of GSH and GSH-SLN-EH was evaluated by the MTT assay. In brief, Fbs cells were plated in 96-well plates (BD Falcon^TM^, BD, Auckland, New Zealand) with a density of 5000 cells/well and cultured to simulate in vitro skin conditions at 37 °C. After 24 h, the DMEM was discarded, and the skin cells were washed with PBS before the cytotoxicity experiment. The filtered GSH solution or GSH-SLN-EH (0.2 mg /mL GSH) was used to treat the Fbs cell lines for 24, 48, and 72 h. Visualization of cell viability was determined by observing the Fbs cells under the microscope (Evos^TM^ XL Core microscope) with a 100× magnification (Thermo Fisher, Waltham, MA, USA). Quantification of cytotoxicity was performed by the MTT assay, in which 20 μL MTT (5 mg/mL) was incubated with cell lines for at least 4 h. Dimethyl sulfoxide (DMSO) was then mixed with the MTT-incubated cells to dissolve the formazan crystals. Cell viability was determined by a microplate UV–Vis spectrophotometer (SpectraMax^®^ Plus, Molecular Devices, San Jose, CA, USA) at 570 nm wavelength.

Cell viability was calculated with the comparison to the untreated control group for each group and at each time point according to Equation (1):

(1) $$V=\frac{A}{A_{0}}100\%$$

where V is the cell viability; *A* is GSH sample absorbance; and *A*_0_ is blank control absorbance.

#### 2.5.3. Antioxidant Activity Evaluation—UVA Irradiation Rescue

The UVA irradiation assay was used to evaluate the antioxidant activity of the GSH solution and GSH-SLN-EH (0.2 mg /mL GSH), respectively. Fbs cells were plated into 96-well plates with the same cell density as the cytotoxicity study and then exposed to UVA irradiation. It was conducted using a wavelength from 320 to 390 nm and a wave peak around 360 nm (Cole-Parmer, Chicago, IL, USA) with a power of 0.75 J/cm^2^. After this UVA irradiation, the GSH solution and GSH-SLN-EH suspension (0.2 mg/mL GSH) samples were added into the flasks in the presence of Fbs and incubated for 1, 2, and 3 days, respectively. Visualization of cell viability was analyzed under an Evos^TM^ XL Core microscope with a magnification of 100×, and quantification of cell viability was determined using the MTT assay.

### 2.6. Ex Vivo Human Skin Evaluation

#### 2.6.1. Skin Tissue Preparation

Full-thickness human skin samples were used for this study. The skin tissues were acquired from the breast reduction of a female patient provided by Middlemore Hospital, Auckland, New Zealand. Approval was obtained from the Human Participants Ethics Committee, University of Auckland (Ref: 010990) for using human skin. Before using skin samples, the additional subcutaneous fat on the surface of the skin was cautiously removed with a scalpel (size 20). The skin was stored at −20 °C in an aluminum foil wrap prior to use.

#### 2.6.2. Franz Diffusion Cell Preparation

Percutaneous permeation and deposition studies were conducted using a Franz diffusion cell (VTC 200, Logan Instruments Corporation, Somerset, NJ, USA). In brief, the receptor compartment of the Franz cells was filled with 12 mL of PBS (pH 5.5). The temperature of the receptor chamber was set and maintained at 34 ± 0.5 °C by a water bath heater and stirred by a magnetic bar at 550 rpm. The donor chamber and receptor chamber were separated by full human skin with SC facing upwards towards the donor chamber (skin tissues were pre-soaked in the PBS (pH 5.5) for 6 h). The effective diffusional area was 1.77 cm^2^. All skin tissues with acceptable integrity were placed in the Franz cells for at least a 1 h equilibration before being used for this study [56,57].

#### 2.6.3. Evaluation of Skin Integrity

Skin integrity was obtained by determining the electrical resistance (ER) of the full-thickness skin tissues with a Millicell-ERS device (Merck, Darmstadt, Germany). The skin ER values crossing the skin surface area of 1.77 cm^2^ were directly read by the Millicell-ERS device. The ER cut-off values for the full-thickness skin tissue in humans should be at least 27.4 kΩ cm^2^ to achieve the minimum skin integrity [58].

#### 2.6.4. Permeation Studies

Aliquots of 1.5 mL of two samples (GSH solution and GSH-SLN-EH) were added to the individual donor chambers of the Franz diffusion cells in triplicates. At predetermined time points (15 min, 30 min, and 1, 2, 3, 6, 9, 12, 24, and 48 h), a known volume (450 µL) of the samples were withdrawn and immediately replaced with the same volume of fresh PBS (pH 5.5). The samples were mixed with 50 μL TCA to precipitate protein and then centrifuged at 15,000 rpm for 10 min. The resulting supernatants were filtered through a 0.45 μm syringe filter and analyzed by the HPLC method.

The cumulative amount (*Q_t_*, μg/cm^2^) of GSH across the skin was calculated by Equation (2) and was plotted with respect to different time points:

(2) $$Q_{t}=\left[V_{r}C_{t}+\sum_{n=0}^{t-1}V_{n}C_{n}\right]\frac{1}{A}$$

whereas *V_r_* is the volume of sample in the receptor chamber of the Franz cells; *C_t_* is the drug concentration at different time intervals; *V_n_* is the volume of withdrawn sample for each time; *C_n_* is the drug concentration of the withdrawn sample at n timepoint; and *A* is the Franz cell’s relative diffusion surface area.

#### 2.6.5. Deposition Studies

##### Stripping of the SC

The skin samples were wiped with methanol ten times, and SC was taken off using the stripping method by an adhesive tape for 15 times (Scotch^®^ Magic tape, 810, 3M center, St Paul, MN, USA). In brief, the skin surface was tapped with adhesive tape for 10 s by applying a 2.5 kg weight. Subsequently, these tapes were rapidly removed from the skin surface with tweezers. This process was repeated 15 times. The resulting tapes with the attached SC were soaked in a mixture of PBS and methanol (5 mL each) for more than 2 h to extract GSH [59].

##### Drug Extraction from Viable Epidermis and Dermis

After tape-stripping SC of the skin, the other parts of the skin were cut into small pieces for extraction. In brief, the resulting skin pieces were collected into a 15 mL centrifuge tube and mixed with 5 mL PBS and 5 mL methanol. The samples were dissociated and homogenized by a T18 Ultra Turrax Homogenizer (IKA^®^, Staufen, Germany) at 25,000 rpm for 10 min. Afterwards, the dissociated samples were mixed with 100 μL TCA to precipitate protein for 1 min and centrifuged at 15,000 rpm for 10 min. The supernatant was filtered and quantified for GSH by HPLC.

Skin fibroblast (Fbs) cells were cultured in DMEM medium, which is composed of 10% FBS, 1% non-essential amino acid (NEAA), and 1% penicillin–streptomycin (antibiotics) with pH values maintained at 7.4 ± 0.2. The cells were incubated in an incubator at 37 °C in the presence of 5% CO_2_. Fbs cells were washed with PBS and cultured with fresh cell medium every three days until the cell confluence reached 80% for further in vitro investigations.

### 2.7. Statistical Analysis

Data analysis of in vitro and ex vivo studies was conducted based on GraphPad Prism^®^ 10.0, and the significance was analyzed by two-way ANOVA (*p* value < 0.05). Tukey’s test method was used to determine the post hoc multiple comparisons for the results of the experimental data.

## 3. Results and Discussion

### 3.1. Analytical Method of GSH

Using the previously established analytical method to quantify the amount of GSH in cell assays, a distinct peak with an adequate retention time at 5.4 min was achieved (more information was shown in the Supplementary Figure S1) [16]. The calibration curve, with an r^2^ value exceeding 0.9998, was established, and the corresponding standard equation (Equation (3)) applicable to the working concentration range of 1 to 100 µg/mL is provided below:

(3) Y=13.042X+17.883

*Y* represents the area under the curve of the HPLC chromatogram, and *X* signifies the concentration of the drug. This equation is utilized for the quantification of GSH in subsequent investigations.

### 3.2. Characterization Studies of GSH-SLN-EH

#### 3.2.1. Morphological Study

**Figure 1:**
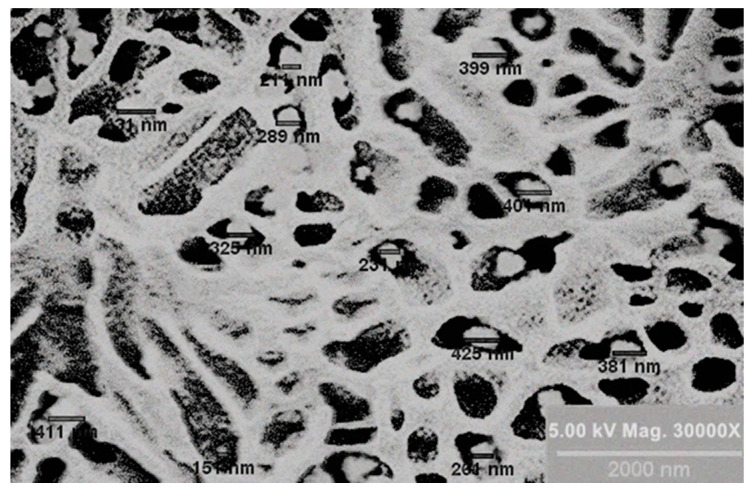
The SEM image of dry GSH-SLN-EH (where the black area is the mesh size of dry cross-linked hydrogels).

The morphology of the dried GSH-SLN-EH (3% (*w*/*v*) Carbopol 971) was characterized using Scanning Electron Microscopy (SEM), as shown in Figure 1. The SEM images reveal that the GSH-SLNs are uniformly distributed, with a size range of 200 to 400 nm, demonstrating an acceptable size distribution within the measured area. These nanoparticles are integrated within the hydrogel system, whose mesh size is approximately 1200 nm, providing adequate space for the nanoparticles to be stored effectively [60]. This optimal structure of the hydrogel ensures the proper encapsulation and sustained release of the nanoparticles, facilitating enhanced topical delivery of GSH for anti-ageing applications.

#### 3.2.2. FTIR Analysis

**Figure 2:**
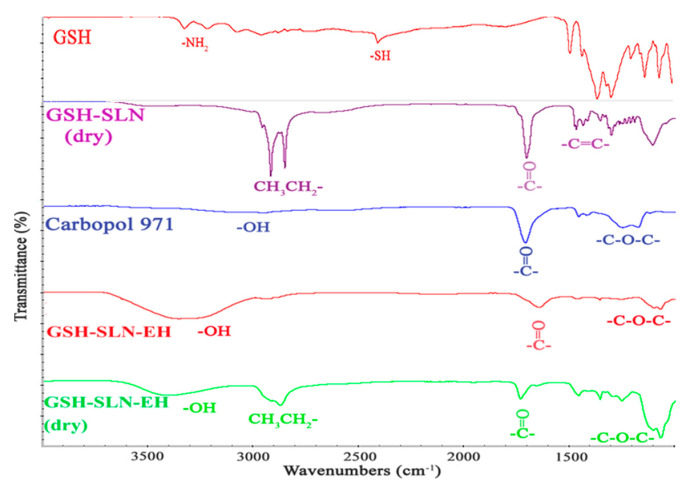
FTIR spectra of GSH, GSH-SLN dry powder, gelling polymers (Carbopol 971), GSH -SLN-EH, and dry GSH-SLN-EH.

The FTIR spectra of GSH, GSH-SLN-EH dried powder, Carbopol 971 powder, GSH-SLNs, and dried GSH-SLNs are presented in Figure 2. The Carbopol 971 spectrum exhibits a broad peak between 3500 and 3000 cm^−1^, corresponding to -OH stretching, a prominent C=O stretching peak at 1636 cm^−1^, and a peak around 1300 to 1200 cm^−1^, which is attributed to the ether linkages (C-O-C) in the acrylate cross-linking groups [61]. For both GSH-SLN-EH and dried GSH-SLN-EH, the characteristic peaks of the GSH-SLNs are significantly diminished, with the spectrum predominantly reflecting the peaks of Carbopol 971. This suggests that the GSH-SLNs are effectively incorporated into the cross-linked structure of the hydrogel, confirming the successful encapsulation of the nanoparticles within the hydrogel matrix.

#### 3.2.3. Texture Profile Analysis

##### Gel Strength

**Figure 3:**
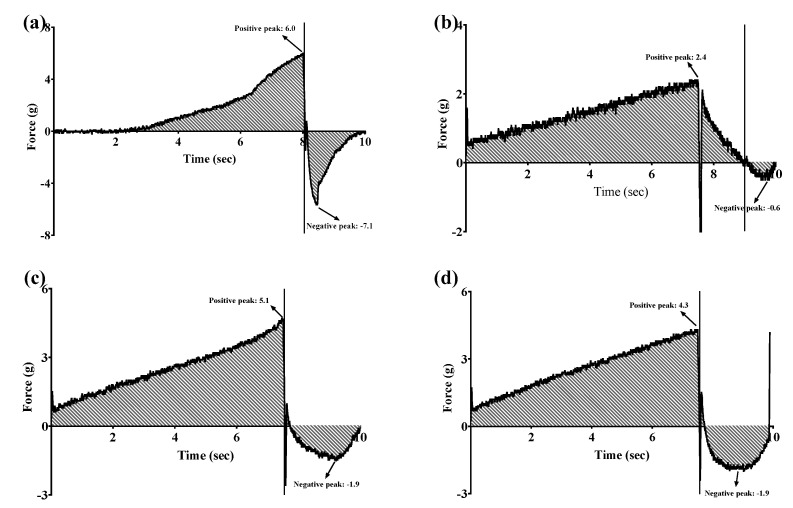
Gel strength (positive peak), adhesion (negative area), and stickiness (negative peak) of (**a**) commercial control gel; (**b**) GSH-SLN-EH with 1% (*w*/*v*) Carbopol 971 inside; (**c**) GSH-SLN-EH with 3% (*w*/*v*) Carbopol 971 inside; and (**d**) GSH-SLN-EH with 5% (*w*/*v*) Carbopol 971 inside.

The gel strengths of three GSH-SLN-enriched hydrogels (GSH-SLN-EHs) with varying concentrations of Carbopol 971 along with a commercial control gel (Merino^®^ 97% Pure Aloe Vera Gel, New Zealand) are presented in Table 1 and Figure 3. The positive peaks and negative peaks in the gel strength test graph represent the gel strength and gel cohesiveness, respectively. The commercial gel exhibited a gel strength of 6.0 g, which was used as the reference standard for gel strength in the development of hydrogels for this study. Among the three formulations tested, the 3% (*w*/*v*) Carbopol 971 hydrogel demonstrated a gel strength most comparable to the commercial control, establishing it as the optimal formulation for this project. Although the 5% Carbopol hydrogel exhibited similar results to the 3% formulation, its higher concentration led to dissolution issues, introducing significant variability that posed challenges for quality control. Consequently, the 3% formulation was selected for its superior consistency and reliability. The decision to establish gel strength requirements based on the commercial gel was due to the absence of standardized parameters for semi-solid dosage forms in the ICH guideline or BP [54,55]. Thus, similarity to the commercial gel served as a benchmark to determine the best gel formulation across all gel characteristics. Additionally, the similarity to the commercial gel enhances the potential for customer acceptability, as the control gel is widely available and popular both in New Zealand and globally.

##### Gel Spreadability

**Figure 4:**
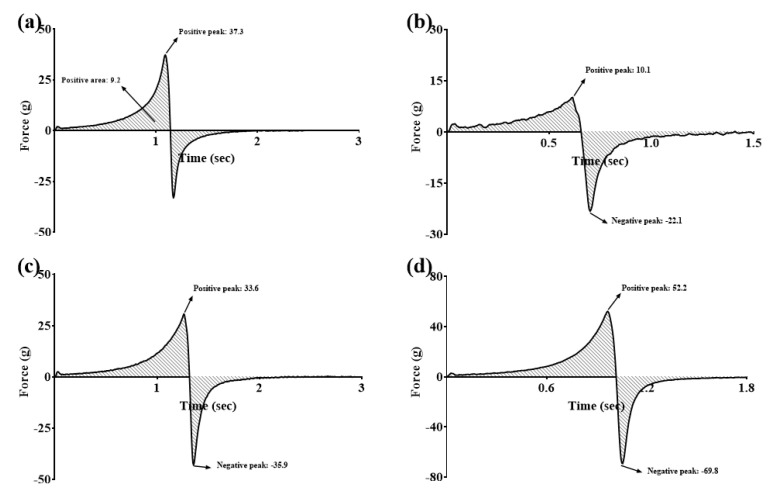
Gel spreadability (positive peak area) of (**a**) commercial control gel; (**b**) GSH-SLN-EH with 1% (*w*/*v*) Carbopol 971 inside; (**c**) GSH-SLN-EH with 3% (*w*/*v*) Carbopol 971 inside; and (**d**) GSH-SLN-EH with 5% (*w*/*v*) Carbopol 971 inside.

The gel spreadability of three GSH-SLN-enriched hydrogels (GSH-SLN-EHs) with varying concentrations of Carbopol 971 along with a commercial control gel (Merino^®^ 97% Pure Aloe Vera Gel, New Zealand) is shown in Table 2 and Figure 4. As detailed in the methods section, the positive peaks in the spreadability test graph correspond to the gel’s spreadability. The 3% (*w*/*v*) Carbopol 971 hydrogel exhibited spreadability properties similar to those of the commercial gel, making it the most suitable formulation for this study. Acceptable spreadability is crucial for optimal application on human skin and ensures that manufacturers can develop appropriate packaging and storage solutions for future use.

In summary, based on the texture profile analysis, the 3% (*w*/*v*) Carbopol hydrogel formulation in GSH-SLN-EH was identified as the optimal choice among the three semi-solid dosage forms due to its similarity in parameters to the commercial gel (Merino^®^ 97% Pure Aloe Vera Gel, New Zealand). To further validate this formulation and gain a deeper understanding of its rheological properties, additional rheological studies were conducted on all three formulations, including the control group, as detailed in the following section.

#### 3.2.4. Rheological Studies

##### Flow Behavior and Thixotropic Properties

**Figure 5:**
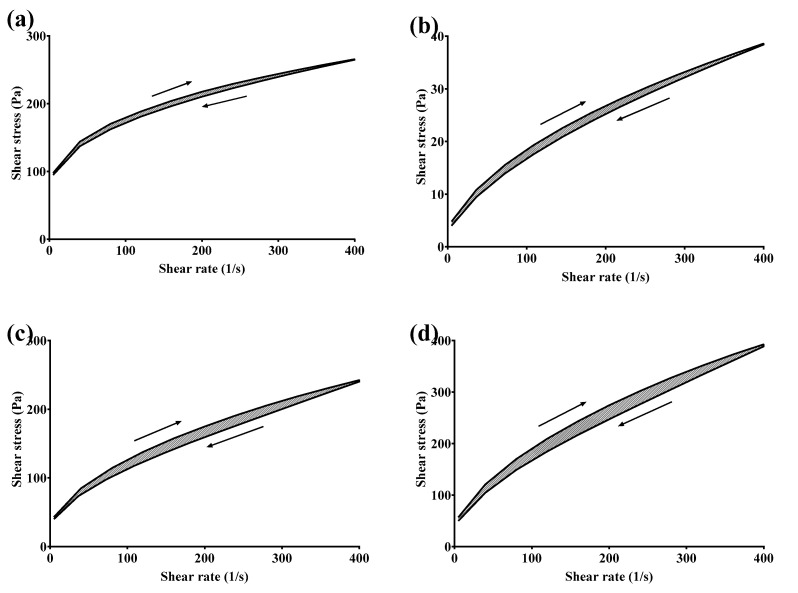
Rheograms of four tested gels. (**a**) Merino^®^ 97% Pure Aloe Vera Gel (control); (**b**) GSH-SLN-EH with 1% (*w*/*v*) Carbopol 971; (**c**) GSH-SLN-EH with 3% (*w*/*v*) Carbopol 971; (**d**) GSH-SLN-EH with 5% (*w*/*v*) Carbopol 971.

**Figure 6:**
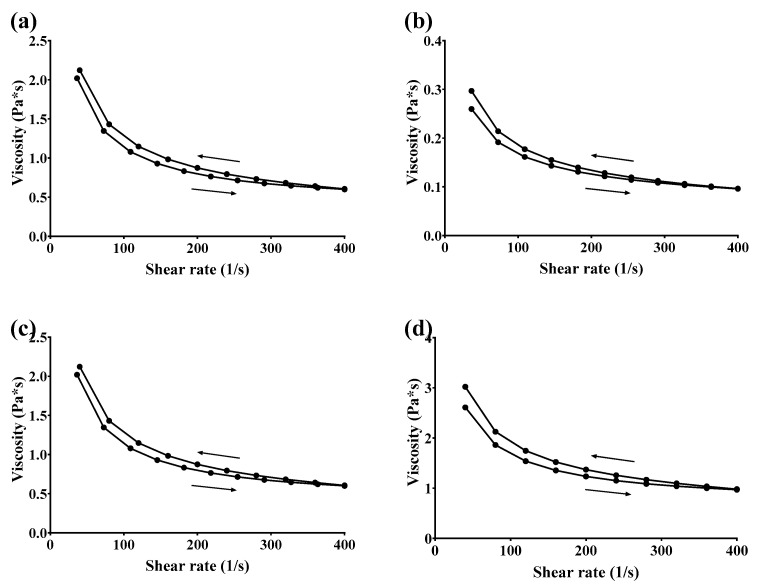
Viscograms of four tested gels. (**a**) Merino^®^ 97% Pure Aloe Vera Gel (control); (**b**) GSH-SLN-EH with 1% (*w*/*v*) Carbopol 971 inside; (**c**) GSH-SLN-EH with 3% (*w*/*v*) Carbopol 971 inside; (**d**) GSH-SLN-EH with 5% (*w*/*v*) Carbopol 971 inside.

Rheological characterization of the GSH-SLN-EHs and the commercial control gel (Merino^®^ 97% Pure Aloe Vera Gel, New Zealand) was performed by plotting shear stress versus shear rate, as shown in Figure 5. The corresponding viscograms in Figure 6 illustrate the viscosity changes as a function of shear rate, revealing distinct rheological behaviors among the four formulations. All three GSH-SLN-EHs demonstrated a proportional increase in shear rate with increasing shear stress, exhibiting a non-linear relationship between these two parameters. The viscograms indicate shear rate-dependent behavior, characteristic of pseudoplastic fluids, a common feature of hydrogel systems. Pseudoplastic fluids are typically polymeric materials that exhibit shear-thinning behavior, wherein the structure of the folded polymers breaks down into unfolded polymers under increased shear stress [62].

Thixotropic behavior was also observed in the rheograms (Figure 5) and viscograms (Figure 6). This behavior was characterized by a gradual increase in shear rate, followed by a decrease back to zero, with the downward curve displaced relative to the upward curve. In shear-thinning systems, the downward curve is typically shifted to the right of the upward curve, indicating lower viscosity at any given shear rate. Thixotropic fluids temporarily lose their secondary polymer structure with increasing shear rate, but this structure is restored when the shear rate decreases. This behavior is beneficial for formulation stability and ensures an optimal profile for skin application, as supported by the literature [62,63].

Based on the shear stress and viscosity results obtained from the rheograms and viscograms of the three candidate hydrogels, the 3% (*w*/*v*) Carbopol hydrogel exhibited the most similar rheological profile to Merino^®^ 97% Pure Aloe Vera Gel. This Carbopol hydrogel demonstrated an acceptable shear stress range of 70 to 250 Pa and a suitable viscosity range of 0.7 to 2.2 Pa·s, aligning with the typical requirements for commercial applications [64,65].

##### Viscoelasticity Properties

The rheological behavior of topical or transdermal formulations significantly influences their spreadability, retention, and contact time on the skin surface. Viscosity and elasticity are essential quality attributes during the early stages of product development. Oscillatory measurements are widely regarded as one of the most effective methods for assessing rheological properties [66].

**Figure 7:**
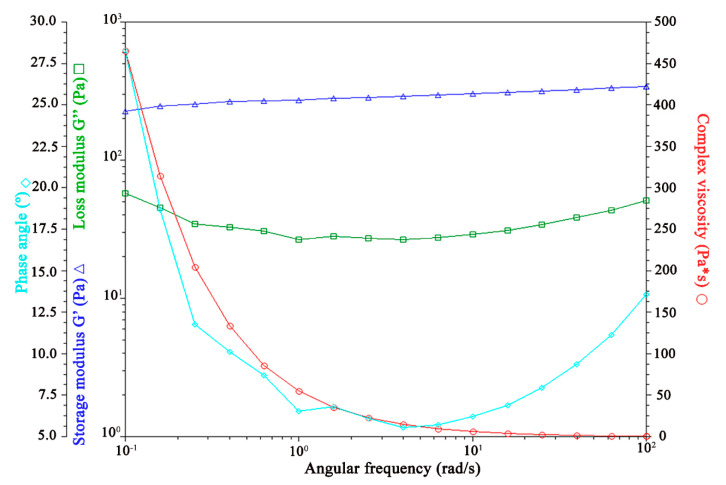
Influence of oscillation rate on both storage and loss modulus in Merino^®^ 97% Pure Aloe Vera Gel.

**Figure 8:**
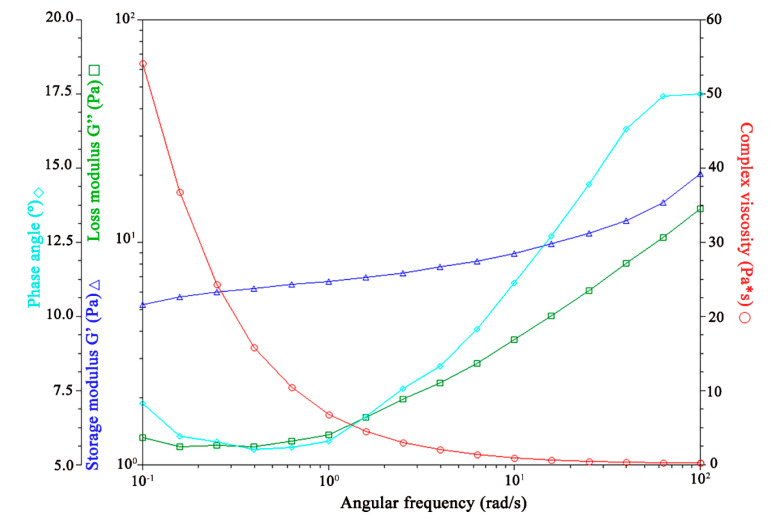
Influence of oscillation rate on both storage and loss modulus in GSH-SLN-EH with 1% (*w*/*v*) Carbopol 971 inside.

**Figure 9:**
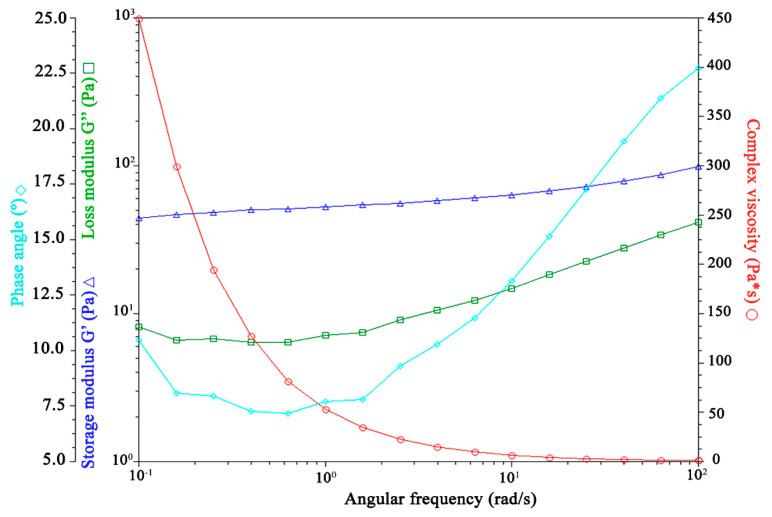
Influence of oscillation rate on both storage and loss modulus in GSH-SLN-EH with 3% (*w*/*v*) Carbopol 971 inside.

**Figure 10:**
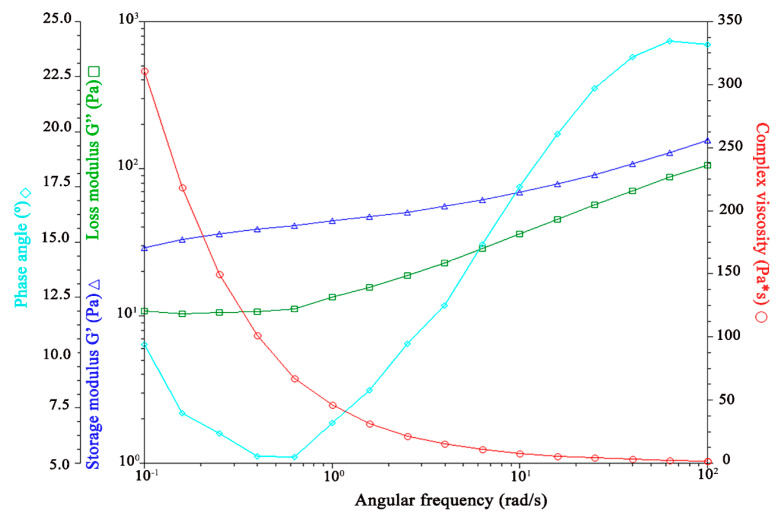
Influence of oscillation rate on both storage and loss modulus in GSH-SLN-EH with 5% (*w*/*v*) Carbopol 971 inside.

Figure 7, Figure 8, Figure 9 and Figure 10 illustrate the results of the oscillatory studies for the three GSH-SLN-EHs and one commercial control gel. The storage modulus (G’) and loss modulus (G”) were measured as a function of angular frequency.

In the control gel, as shown in Figure 7, the storage modulus (G’) consistently exceeds the loss modulus (G”) across all frequencies, indicating a strong elastic behavior and a predominantly solid-like structure.

In the three GSH-SLN-EHs shown in Figure 8, Figure 9 and Figure 10, similar trends to the control group were observed, with the storage modulus (G’) consistently exceeding the loss modulus (G”) at all frequencies. However, compared to the control group, narrower gaps between G’ and G” were observed at higher frequencies for all three Carbopol hydrogels. These narrower gaps indicate a transition region where G’ approaches G”, suggesting a shift from solid-like to liquid-like behavior. This transition is beneficial for topical formulations, as it enables better spreadability upon application to the skin. The formulation’s ability to transition from solid-like to liquid-like behavior is critical for optimal skin application. Based on these results, all three Carbopol hydrogels demonstrate superior viscoelasticity properties for topical delivery when compared to Merino^®^ 97% Pure Aloe Vera Gel.

In summary, the rheological characterization studies of the GSH-SLN-EHs reveal that the optimal formulation is the GSH-SLN-EH containing 3% and 5% (*w*/*v*) Carbopol 971. However, due to the dissolution issue of 5%(*w*/*v*) Carbopol 971, only 3% (*w*/*v*) Carbopol 971 was selected for further characterization (more support information was shown in the Supplementary Figure S6). This formulation exhibits thixotropic pseudoplastic behavior, demonstrating excellent gel strength (5.1 g) and gel spreadability (33.6 g·s) and acceptable viscoelastic properties, making it highly suitable for efficient topical delivery.

#### 3.2.5. In Vitro Drug Release Studies

**Figure 11:**
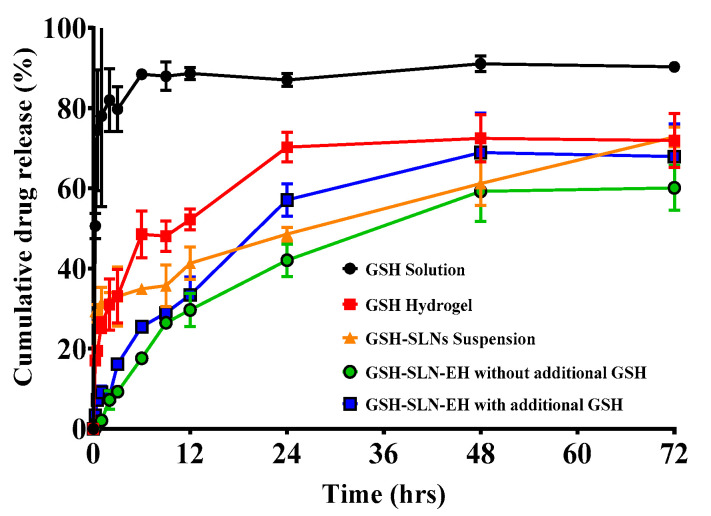
Release profiles of all samples of GSH, namely GSH solution, GSH-loaded hydrogels, GSH-SLN suspension, and GSH-SLN-EH with and without additional GSH in hydrogels (Mean ± S.D. n = 3).

**Figure 12:**
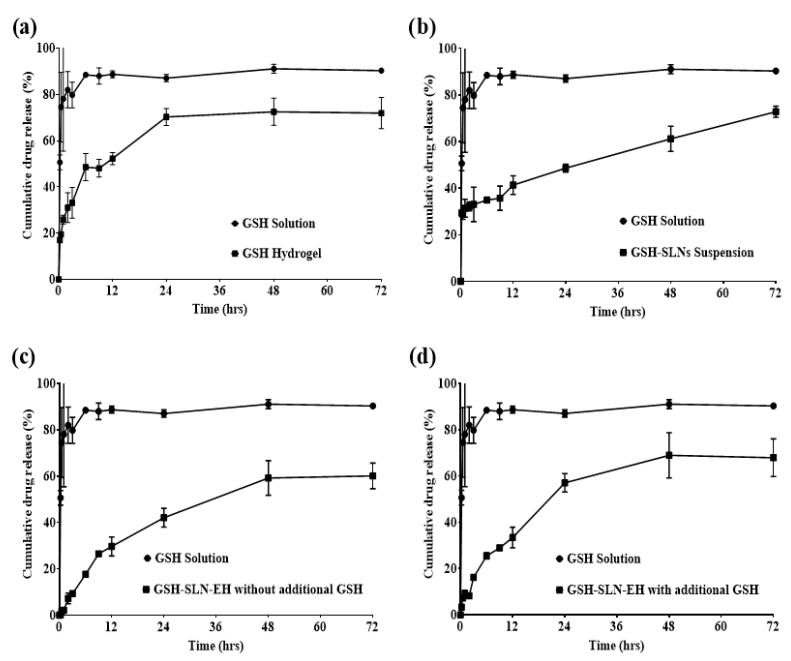
Release profiles of (**a**) GSH hydrogels; (**b**) GSH-SLN suspension; (**c**) GSH-SLN-EH without additional GSH in hydrogels; (**d**) GSH-SLN-EH with additional GSH in hydrogels compared with GSH solution (Mean ± S.D. n = 3).

In vitro release studies are a crucial aspect of formulation characterization and quality control in both pharmaceutical and cosmetic research, as they ensure that bioactive compounds are released from the formulation at the desired rate. The release profiles of the GSH solution, GSH-loaded hydrogels, GSH-SLN suspension, and GSH-SLN-EH (with and without additional GSH in the hydrogels) were evaluated over a 72 h period (Figure 11). The resulting data were analyzed and fitted to various release kinetics models, as depicted in Figure 12.

GSH solution: As shown in Figure 12a, GSH was released at a rate exceeding 90% within 5 h, highlighting the need for the development of a controlled-release formulation. Controlled release is essential for topical applications, as it allows for delayed release and sustained delivery of the compound to the skin, enhancing efficacy and extending patient protection over time [67].

GSH-loaded hydrogels: Figure 12a shows that the release profile of GSH from the hydrogel formulation was significantly modified compared to the free GSH solution. A burst release of approximately 20% of GSH occurred within the first hour, followed by a sustained release for more than 24 h. At the end of 72 h, about 75% cumulative release of GSH was observed. The observed loss of GSH may be attributed to hydrolysis and degradation of GSH within the water-based hydrogel matrix.

GSH-SLN suspension: Figure 12b demonstrates a sustained-release profile from the GSH-SLN suspension formulation. Approximately 20% of GSH was released as a burst within the first hour due to the presence of unentrapped free GSH in the suspension medium. This was followed by a prolonged release lasting more than 72 h, with about 75% of the cumulative release detected by the end of the study. Notably, the release curve continued to increase beyond 72 h, indicating that some GSH remained trapped within the solid lipid nanoparticles (SLNs), contributing to the extended-release profile.

GSH-SLN-EH without additional GSH in hydrogels: Figure 12c shows that GSH-SLN-EH without additional GSH in the hydrogels exhibited a controlled-release profile without a burst release. A sustained release over 72 h was observed, with approximately 65% of GSH released by the end of the study, demonstrating efficient long-term delivery.

GSH-SLN-EH with additional GSH in hydrogels: In Figure 12d, GSH-SLN-EH with additional GSH in the hydrogels exhibited a modified-release profile compared to the free GSH solution. A burst release of approximately 10% occurred within the first hour due to the rapid release of free GSH from the hydrogels. Following this, a sustained-release profile was observed for more than 72 h, with approximately 70% cumulative release at the end of the study. This extended, sustained-release profile offers significant advantages for maintaining therapeutic drug levels in the skin without the need for frequent reapplication, ensuring prolonged efficacy [68].

The data obtained from the in vitro drug release studies were analyzed using various kinetic models to gain insights into the mechanism and kinetics of drug release across different formulations. The kinetic parameters derived from the modeling are presented in Table 3 (more information was shown in the Supplementary Figures S2–S4). To determine the most appropriate release models, linear regression analysis (R^2^) was employed to evaluate the goodness of fit, with the best-fitting models being selected based on the highest R^2^ values.

GSH-loaded hydrogels: As depicted in detail in Table 3, the drug release data for GSH-loaded hydrogels best fit the Korsmeyer–Peppas model, with an R^2^ value of 0.974. The release exponent (n) value of 0.29, which is less than 0.43, indicates a Fickian diffusion-mediated release mechanism. Fickian diffusion is characteristic of polymeric dosage forms, which aligns with the nature of the hydrogel systems used in this study, primarily composed of polymers [69,70,71]. Hence, the release mechanism of GSH-enriched hydrogels follows a Fickian diffusion pattern as described by the Korsmeyer–Peppas model.

GSH-SLN suspension: As shown in Table 3, the release profile of the GSH-SLN suspension best fits the first-order kinetic model, with an R^2^ value of 0.908. The first-order model is typically applicable to highly soluble compounds released from porous matrices, such as the lipid nanoparticles (SLNs) in this study, which are considered nanoparticulate porous matrices [72]. The release rate in this model depends on the concentration of the drug remaining in the release medium, suggesting that the drug release from GSH-SLNs follows a first-order release mechanism [73].

GSH-SLN-EH without additional GSH in hydrogels: The release profile of GSH-SLN-EH without additional GSH in hydrogels, as shown in Table 3, best follows the Higuchi model, with an R^2^ value of 0.971. The Higuchi model is typically used to describe the release of soluble active ingredients from insoluble matrices, which corresponds to the nature of GSH-SLNs (insoluble base) incorporated into semi-solid hydrogel matrices in this formulation [69,74]. Thus, the drug release mechanism for this formulation follows Higuchi diffusion.

GSH-SLN-EH with additional GSH in hydrogels: As illustrated in Table 3, the release profile of GSH-SLN-EH with additional GSH in hydrogels best fits the Korsmeyer–Peppas model, with an R^2^ value of 0.965. The release exponent (n) value of 0.54 falls within the range of 0.43 to 0.85, indicating a non-Fickian release mechanism or a coupled diffusion and erosion process [69,70,71]. This suggests a more complex release mechanism, involving both diffusion and erosion processes, which warrants further investigation for a more detailed understanding of the release behavior.

In summary, controlled-release profiles were observed in GSH-loaded hydrogels, GSH-SLN suspension, and GSH-SLN-EH formulations (both with and without additional GSH). In contrast, the GSH solution exhibited a rapid-burst release, with over 90% of the drug released within 5 h. The release mechanisms of the formulations were characterized as follows: Fickian diffusion-mediated release for GSH-enriched hydrogels, first-order release for GSH-SLN suspension, Higuchi diffusion for GSH-SLN-EH without additional GSH, and non-Fickian release (coupled diffusion and erosion) for GSH-SLN-EH with additional GSH. These findings highlight the diverse release kinetics and mechanisms for each formulation, supporting their potential for controlled, sustained topical delivery of GSH.

### 3.3. In Vitro Cell Culture Investigation

#### 3.3.1. Cytotoxicity Study

**Figure 13:**
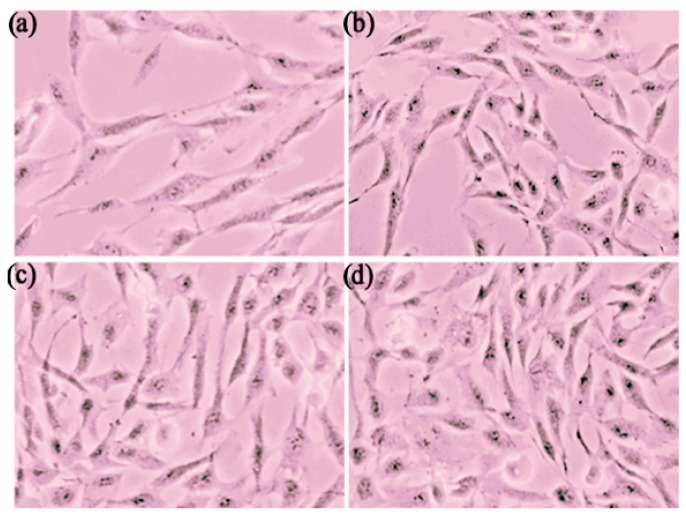
The microscopic images of Fbs cells after 72 h. (**a**) Fbs cells without any GSH formulations; (**b**) Fbs cells treated with GSH solution; (**c**) Fbs cells treated with GSH-SLN suspension; (**d**) Fbs cells treated with GSH-SLN-EH.

Cytotoxicity studies were performed to evaluate the effect of L-glutathione (GSH) and its formulations on the cell viability of fibroblasts (Fbs), with three different GSH formulations: GSH solution, GSH-loaded solid lipid nanoparticle (GSH-SLN) suspension, and GSH-loaded solid lipid nanoparticle-enriched hydrogels (GSH-SLN-EH). Microscopic images of Fbs cells treated with these formulations for 72 h are shown in Figure 13. The results demonstrate an increase in the number of viable Fbs cells across all formulations, indicating that none of the GSH formulations exhibited cytotoxic effects. The formulations provided cytoprotective effects, preventing Fbs cell death.

**Figure 14:**
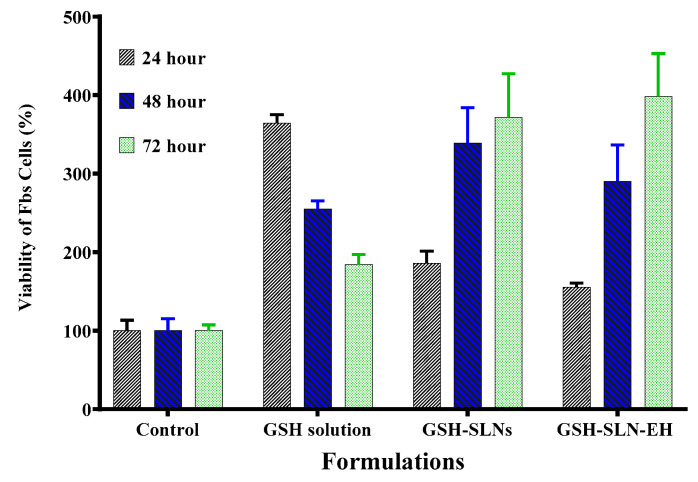
Effect of three formulations of GSH on the Fbs cell viability. Cells were incubated for 24, 48, and 72 h; control is cell culture medium only without any treatment of GSH (*p* value ˂ 0.05) (Mean ± SD, n = 5).

Figure 14 further illustrates the non-cytotoxicity of all GSH formulations at three different time intervals (24, 48, and 72 h). These findings correlate with the results from preformulation studies, where GSH was shown to promote cell growth. Based on the distinct release profiles observed for each formulation, cell viability increased in the GSH-SLN and GSH-SLN-EH formulations, while it decreased with the GSH solution. After 72 h, the GSH-SLN-EH group demonstrated a 370% increase in cell viability, which is 3.7-fold higher than the control group. This enhanced cell viability can be attributed to two factors: first, the prevention of cell death in the GSH-SLN-EH group compared to the control group and, second, the antioxidant properties of GSH, which scavenge free radicals and promote cell growth [75]. These results confirm the safety and efficacy of GSH-SLN-EH formulations in enhancing cell viability.

#### 3.3.2. Antioxidant Activity Evaluation—UVA Irradiation Rescue

**Figure 15:**
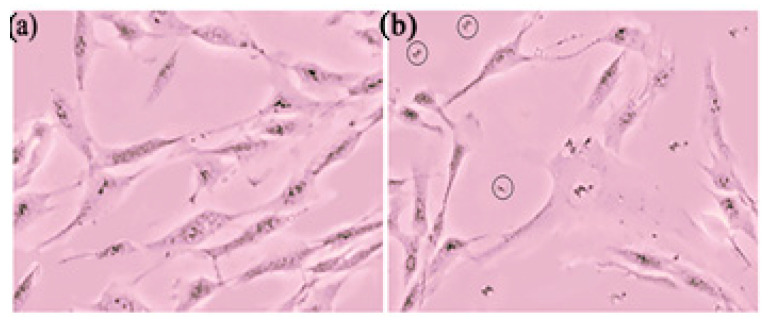
The micrographs of two control groups of Fbs cells, treated without (**a**) and with (**b**) UVA irradiation after culture for 72 h; the black circles in figure b represent dead Fbs cells.

**Figure 16:**
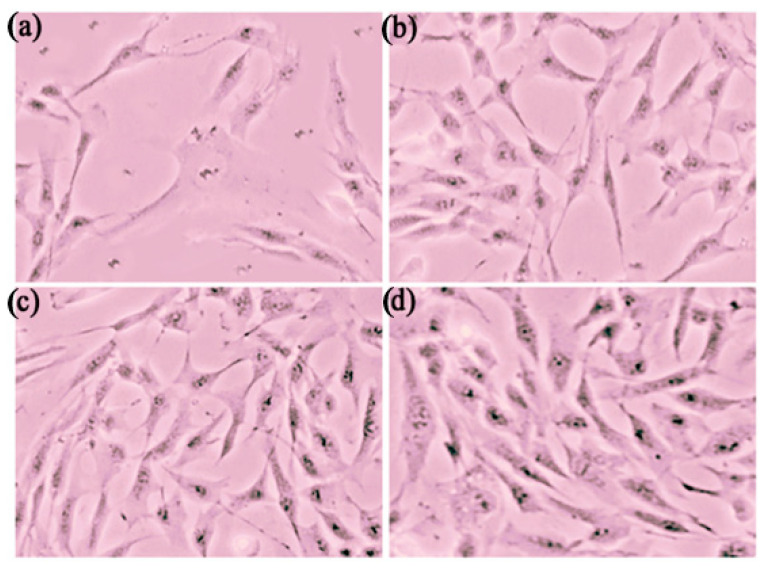
The micrographs of Fbs cells treated with a half hour of UVA irradiation after 72 h. (**a**) Fbs cells without any GSH formulations; (**b**) Fbs cells treated with GSH solution; (**c**) Fbs cells treated with GSH-SLN suspension; (**d**) Fbs cells treated with GSH-SLN-EH.

UVA irradiation is known to generate excessive reactive oxygen species (ROS) in the skin, leading to oxidative stress and contributing to skin aging and other skin diseases [76]. As reported by Starr et al., skin exposed to UVA radiation can overwhelm the body’s endogenous antioxidants, causing cellular damage. UVA radiation penetrates the epidermis and dermis, resulting in oxidative stress and ROS generation. Several studies have used UVA irradiation as an oxidative stress model to investigate the antioxidant activity of various drugs [77,78]. Cell viability, which can alleviate the toxic effects of ROS, decreases when ROS production exceeds the protective capacity of antioxidants [79]. The antioxidant effects of GSH in the three formulations were evaluated by comparing two control groups: one without GSH treatment and one with GSH treatment but no UVA irradiation. As shown in Figure 15 and Figure 16, after 30 min of UVA irradiation for 72 h, the Fbs cells exhibited approximately 60% viability compared to the untreated control group.

**Figure 17:**
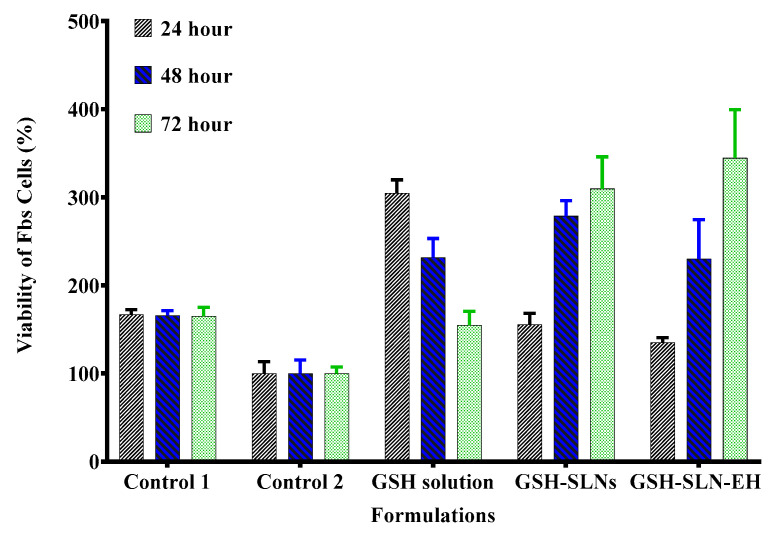
Cytoprotective effects of GSH formulations at three time intervals (24, 48, and 72 h). The control group (2) was exposed to UVA irradiation without GSH treatment. Cell viability increased significantly for all GSH formulations (*p* value < 0.05) (Mean ± SD, n = 5).

As shown in Figure 17, all GSH formulations demonstrated cytoprotective effects at three different time intervals (24, 48, and 72 h). Similar to the cytotoxicity studies, the GSH-SLN and GSH-SLN-EH formulations exhibited a controlled-release profile, enhancing cell viability over time, from 24 to 72 h, compared to the GSH solution. After 72 h of treatment with the GSH-SLN suspension, Fbs cell viability was rescued to around 310% of the control group, with a 3.1-fold increase in viability. Furthermore, Fbs cells treated with GSH-SLN-EH showed a cell viability increase ranging from 150% at 24 h to 350% at 72 h. The GSH-SLN-EH group exhibited a higher cell viability at the 72 h mark, which can be attributed to its superior controlled-release profile.

### 3.4. Ex Vivo Human Skin Evaluation Studies

#### 3.4.1. Evaluation of Human Skin Integrity

The integrity of human skin can be evaluated using various techniques, such as (1) measuring the flux of standard dye across the skin [80], (2) assessing trans-epidermal water loss (TEWL) [81], and (3) monitoring the skin’s electrical resistance (ER) [82]. In this study, ER measurement was selected due to its accuracy and efficiency compared to other methods, which may interfere with skin integrity by using dyes or water that could cause swelling or disruption of the stratum corneum (SC) [83,84]. The ER value is inversely proportional to the area of skin in contact with the electrode, making it a practical method for evaluating skin health. Based on the research by Davies et al. [85], ER data were collected for over 30 min post-skin equilibration, with results presented in kΩ·cm^2^. The average ER value for the full-thickness human skin in this study was 28.4 ± 1.8 kΩ·cm^2^, which meets the threshold of 27.4 kΩ·cm^2^ suggested by Hasler-Nguyen et al. [85,86].

#### 3.4.2. Permeation Studies

**Figure 18:**
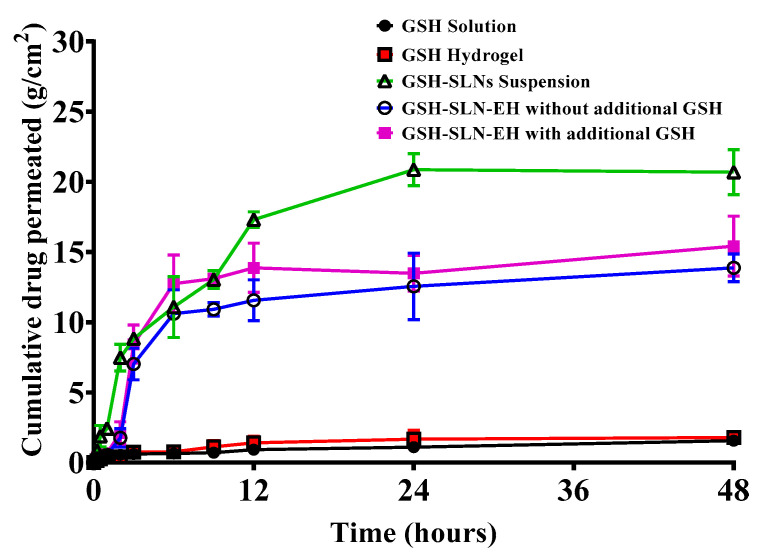
Cumulative permeation data for GSH solution, GSH-SLN, GSH-hydrogel, and GSH-SLN-EH formulations over 48 h (Mean ± S.D., n = 3).

Five formulations, GSH solution, GSH-SLNs, GSH-hydrogels, and GSH-SLN-EH (with and without additional GSH in hydrogels), were tested for ex vivo skin permeation using an equivalent concentration of 0.2 mg/mL GSH. The cumulative amount of GSH that permeated through the skin after 24 and 48 h is shown in Figure 18 and Table 4. Notably, the amount of GSH permeated from the GSH-SLN and GSH-SLN-EH formulations increased over the 48 h period. Pure GSH solution and GSH-hydrogels exhibited significantly lower permeation (1.6 ± 0.4 and 1.8 ± 0.2 μg/cm^2^, respectively) compared to the GSH-SLN (20.7 ± 1.6 μg/cm^2^) and GSH-SLN-EH formulations. GSH-SLNs demonstrated the highest permeation, attributed to a faster drug release rate. The permeation from the GSH-SLN-EH formulations showed a steady increase, reaching 13.9 ± 1.0 μg/cm^2^ and 15.4 ± 2.1 μg/cm^2^ for the formulations with and without additional GSH in the hydrogels, respectively. The GSH-SLN-EH formulation showed an 8.5-fold increase, and GSH-SLNs demonstrated a 12-fold increase in permeation compared to the GSH solution. The higher viscosity of the hydrogel limited initial permeation; however, it provided protective encapsulation for the GSH-SLNs, allowing for a sustained permeation effect over time. With an extended study duration of up to 7 days, the hydrogel formulation is anticipated to outperform the SLNs alone in penetration efficiency due to its prolonged release capabilities.

#### 3.4.3. Deposition Studies

The epidermis and dermis are crucial for the pharmacological efficacy of actives in topical formulations. Effective topical drug delivery aims to penetrate the skin barrier, reaching the viable epidermis and dermis layers. To assess the impact of the different formulations on skin deposition, it is essential to evaluate the amount of drug deposited in the epidermal and dermal layers, excluding the stratum corneum (SC).

The tape-stripping method was successfully employed for SC removal, a technique widely recognized in dermatological research for its efficiency in assessing drug penetration [87]. According to previous studies, at least fifteen tape strips are sufficient for complete removal of the SC layer, with the skin surface appearing glistening, indicating that most of the SC has been removed [87,88,89]. This method’s simplicity and reproducibility make it an ideal choice for studying the penetration and deposition behaviors of topical formulations [90].

**Figure 19:**
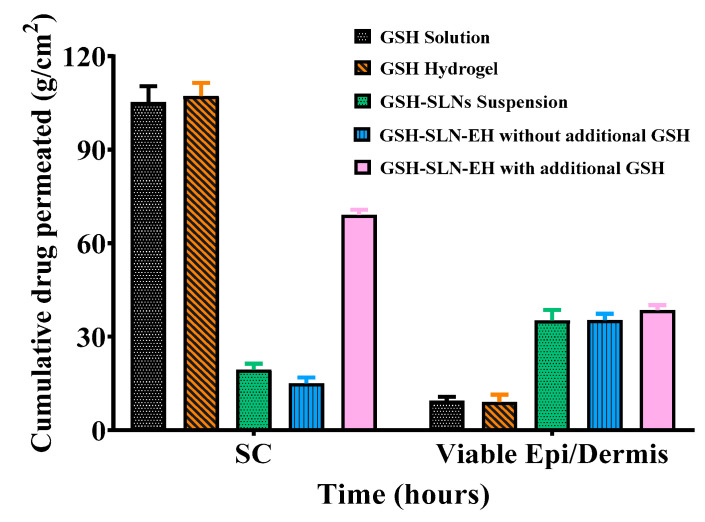
Human skin deposition of GSH in SC and epi/dermis layers for five formulations after 48 h: GSH solution, GSH-SLNs, GSH-hydrogels, and GSH-SLN-EH with and without additional GSH in hydrogels, respectively (Mean ± S.D., n = 3).

This study focused on the deposition of GSH in both the SC and the viable epidermis/dermis (epi/dermis) layers after 48 h of application with five different formulations in the presence of the same drug concentration (i.e., GSH solution, GSH-SLNs, GSH-hydrogels, and GSH-SLN-EH with and without additional GSH in hydrogels). As shown in Figure 19 and Table 5, the amount of GSH deposited in the epi/dermis was enhanced in GSH-SLNs and GSH-SLN-EH when compared with the GSH solution and hydrogels. In the case of the GSH solution, the majority of the GSH remained in the SC layer, with a measured amount of 105.3 ± 5.0 µg/cm^2^, while only 9.5 ± 1.2 µg/cm^2^ was found in the epi/dermis. Similarly, the GSH-hydrogels showed comparable results, with 107.2 ± 4.2 µg/cm^2^ in the SC and only 8.07 ± 2.3 µg/cm^2^ in the epi/dermis, indicating limited skin penetration due to the hydrophilic nature of GSH. In contrast, GSH-SLNs demonstrated a significant improvement in drug penetration. The amount of GSH in the epi/dermis layers was 35.3 ± 3.3 µg/cm^2^, a 3.7-fold increase compared to the GSH solution. This enhancement is likely attributed to the nanometer-scale size of the SLNs, which allows for better penetration through the SC barrier, as evidenced by the deposition of 19.4 ± 1.9 µg/cm^2^ in the SC. The GSH-SLN-EH formulation, with and without additional GSH in the hydrogel, showed even greater penetration efficiency. For GSH-SLN-EH without additional GSH, the GSH deposited in the epi/dermis was 35.4 ± 2.0 µg/cm^2^, similar to the SLNs alone but with reduced SC deposition (15.0 ± 1.8 µg/cm^2^). When additional GSH was added to the hydrogel, the total GSH deposition in the SC layer increased to 69.1 ± 1.7 µg/cm^2^, while the epi/dermis deposition reached 38.5 ± 1.6 µg/cm^2^. Despite the higher SC deposition, the enhanced delivery to the deeper layers of the skin was still significant.

These findings suggest that the use of SLN-based hydrogels significantly improves GSH penetration into the viable skin layers compared to traditional GSH solutions or hydrogels. This enhancement is likely due to a combination of factors, including the small particle size of the SLNs, the use of penetration-enhancing polymers like chitosan and Carbopol, and the rheological properties of the hydrogel formulation, which promote better adhesion to the skin and prolonged contact time. GSH-SLN-EH formulations demonstrate superior penetration into the epi/dermis layers and offer a promising approach for enhancing the topical delivery of anti-ageing agents like L-glutathione. This study provides valuable insights into the mechanisms of enhanced drug delivery through the use of solid lipid nanoparticles and hydrogels, highlighting their potential for improving the efficacy of topical treatments.

Several studies have demonstrated that nanometer-sized carrier systems can significantly enhance drug penetration across the stratum corneum (SC) and improve drug deposition in the skin layers [91,92]. A suitable dispersing medium, such as polymeric semi-solid dosage forms, further aids the application of these carrier systems onto the skin, enhancing drug penetration [93]. However, the mechanisms underlying the enhanced performance of such novel delivery systems remain complex and are not fully understood. Based on previous research, we propose four possible mechanisms for the enhanced topical delivery of GSH using the GSH-SLN-enriched hydrogel (GSH-SLN-EH) formulation:

1. Drug Loading Capacity and Carrier Structure: The first mechanism involves the high drug loading capacity and entrapment efficiency of solid lipid nanoparticles (SLNs), which generate large concentration gradients. These gradients facilitate efficient drug delivery to the skin. The solid lipids, with GSH as the internal phase, are surrounded by surfactants that create a unique structure, enabling SLNs to overcome the skin barrier, which is primarily lipid-based. The particle size of less than 400 nm is optimal for crossing the SC barrier, enabling the delivery of GSH into deeper epidermal and dermal layers [94].

2. Penetration Enhancement by Polymers: The second mechanism pertains to the use of penetration-enhancing polymers in the drug delivery system. Chitosan and Carbopol 971 were used as polymers in the SLNs and hydrogels of the GSH-SLN-EH formulation, respectively. Both of these polymers have been recognized as effective skin penetration enhancers [94,95]. Chitosan has been shown to improve the permeability of the skin by enhancing the fluidity of the lipid bilayers, while Carbopol improves the gel’s adhesive properties, prolonging skin contact.

3. Surfactant-Mediated Enhancement: A third mechanism involves the action of surfactant molecules, which play a significant role in topical drug delivery. Surfactants, particularly in their monomeric form, can cross the SC and act as penetration enhancers by temporarily disrupting the lipid bilayers of the SC. This disruption increases the diffusion of the active ingredient and enhances the partitioning of the drug across the skin barrier. Surfactants lower the barrier resistance, making the skin more permeable to the drug.

4. Rheological Properties of Hydrogels: The fourth mechanism is related to the rheological properties of the hydrogel. Hydrogels are known for their low interfacial tension, which improves surface contact between the formulation and the skin. This property is particularly beneficial for penetrating areas such as skin wrinkles and microscopic gaps. The enhanced adhesion of hydrogels to the skin surface prolongs the contact time, allowing for better absorption and improved drug delivery.

In summary, the GSH-SLN-EH formulation demonstrates a significant enhancement in penetration compared to pure GSH solutions and hydrogels. At 48 h post-application, the SLN-enriched hydrogel formulation exhibited superior drug deposition in the deeper epidermal and dermal layers, indicating its potential for effective anti-ageing treatments. The combination of optimized particle size, penetration enhancers, surfactants, and favorable rheological properties results in a formulation that enhances both skin penetration and drug bioavailability. These findings underscore the potential of GSH-SLN-EH for improving the delivery and efficacy of anti-ageing agents in topical treatments.

## 4. Conclusions

This research developed a novel delivery system incorporating solid lipid nanoparticles (SLNs) enriched in hydrogels (GSH-SLN-EH) to enhance the topical delivery of L-glutathione (GSH) for combating skin ageing caused by oxidative stress. As a potent antioxidant, GSH protects against reactive oxygen species (ROS) but faces challenges in topical delivery due to its hydrophilic nature and skin barrier resistance. Using a double-emulsion method, we encapsulated GSH in SLNs, dispersed them into Carbopol hydrogel, and optimized the system for stability, skin adhesion, and controlled release. The GSH-SLN-EH exhibited favorable mechanical properties, thixotropic behavior, and enhanced skin spreadability. It demonstrated controlled GSH release, antioxidant protection against UVA-induced cellular damage in fibroblasts, and a three-fold improvement in skin penetration and retention over standard formulations. With its stability, sustained release, and superior skin permeation, the GSH-SLN-EH offers a promising anti-ageing formulation and potential applications in wound healing and inflammatory skin diseases.

## Figures and Tables

**Table 1:** Gel strength, adhesion, and stickiness of three GSH-SLN-EHs with different Carbopol concentrations and one commercial control gel (Mean ± S.D. n = 3).

Items	Control Gel	1% Carbopol	3% Carbopol	5% Carbopol
Gel strength (g)	6.0 ± 0.5	2.4 ± 1.2	5.1 ± 0.5	4.3 ± 0.9
Adhesion (g·s)	6.2 ± 0.6	0.5 ± 1.2	2.5 ± 0.6	1.9 ± 1.0
Stickiness (g)	7.1 ± 0.8	0.6 ± 1.1	1.9 ± 0.5	1.9 ± 1.0

**Table 2:** Gel spreadability of three GSH-SLN-EH s with different Carbopol concentrations and one commercial control gel (Mean ± S.D. n = 3).

Items	Control	1% Carbopol	3% Carbopol	5% Carbopol
Spreadability (g·s)	34.3 ± 2.2	10.1 ± 3.1	33.6 ± 1.9	52.2 ± 5.9

**Table 3:** Release kinetic parameters in different models (* the best R^2^ for modelling fit).

Samples	Zero Order		First Order		Higuchi		Korsmeyer–Peppas		
	R^2^	k_0_	R^2^	k_1_	R^2^	k_k_	R^2^	n	k_h_
GSH-gel	0.613	0.812	0.729	0.007	0.848	8.168	0.974 *	0.29	1.407
GSH-SLN	0.755	0.689	0.908 *	0.908	0.832	6.276	0.856	0.15	1.485
EH	0.844	0.901	0.908	0.006	0.971 *	8.255	0.884	0.97	0.320
EH and GSH	0.811	0.987	0.878	0.007	0.964	9.151	0.965 *	0.54	0.926

GSH-gel is free GSH dispersed in hydrogels; EH is GSH-SLN-EH without additional GSH in hydrogels; EH and GSH is GSH-SLN-EH with additional GSH in hydrogels.

**Table 4:** Summary of permeation study across human skin in the presence of GSH solution, GSH-SLNs, GSH-hydrogels, and GSH-SLN-EH with and without additional GSH in hydrogels, respectively, at 24 and 48 h (Mean ± S.D., n = 3).

Formulations	Cumulative amount of GSH at 24 h (µg/cm^2^)	Cumulative Amount of GSH at 48 h (µg/cm^2^)
GSH solution	1.1 ± 0.2	1.6 ± 0.4
GSH hydrogels	1.7 ± 0.6	1.8 ± 0.2
GSH-SLNs	20.9 ± 1.2	20.7 ± 1.6
GSH-SLN-EH	12.6 ± 2.4	13.9 ± 1.0
EH and GSH	13.5 ± 1.3	15.4 ± 2.1

**Table 5:** GSH deposited in SC and epi/dermis layers for five formulations after 48 h: GSH solution, GSH-SLNs, GSH-hydrogels, and GSH-SLN-EH with and without additional GSH in hydrogels, respectively (Mean ± S.D., n = 3).

Formulations	SC Deposition of GSH After 48 h (µg/cm^2^)	Epi/Dermis Deposition of GSH After 48 h (µg/cm^2^)
GSH solution	105.3 ± 5.0	9.5 ± 1.2
GSH gel	107.2 ± 4.2	8.07 ± 2.3
GSH-SLNs	19.4 ± 1.9	35.3 ± 3.3
GSH-SLN-EH	15.0 ± 1.8	35.4 ± 2.0
EH and GSH	69.1 ± 1.7	38.5 ± 1.6

## Data Availability

The original contributions presented in this study are included in the article/Supplementary Material. Further inquiries can be directed to the corresponding authors.

## Supplementary Materials

The following supporting information can be downloaded at: https://www.mdpi.com/article/10.3390/pharmaceutics17010004/s1, Figure S1: GSH HPLC chromatogram; Figure S2: GSH loaded hydrogels release profiles modelling into four release mathematical models (* the best R2 for model selection);.Figure S3: GSH-SLNs suspension release profiles modelling into four release mathematical models (* the best R2 for model selection); Figure S4: GSH-SLN-EH without additional GSH in hydrogels release profiles modelling into four release mathematical models (* the best R2 for model selection); Figure S5: GSH-SLN-EH with additional GSH in hydrogels release profiles modelling into four release mathematical models (* the best R2 for model selection); Figure S6: Release profiles of GSH solution, GSH loaded hydrogels without SLNs, GSH-SLN-EH with 3% Carbopol and 5% Carbopol (Mean ± S.D. n = 3); Table S1: Gel strength, adhesion and stickiness of 3% Carbopol control gel without SLNs loaded (Mean ± S.D. n = 3).
