# Supplementary material for: Innovative Solid Lipid Nanoparticle-Enriched Hydrogels for Enhanced Topical Delivery of L-Glutathione: A Novel Approach to Anti-Ageing
Source: Pharmaceutics. 2024 Dec 24;17(1):4. doi: 10.3390/pharmaceutics17010004 (PMC11768106; doi:10.3390/pharmaceutics17010004)
Supplement: Supplementary file 1 [file pharmaceutics-17-00004-s001.zip › pharmaceutics-3382448-supplementary.pdf]

pharmaceutics

MDPI

Supplementary Materials

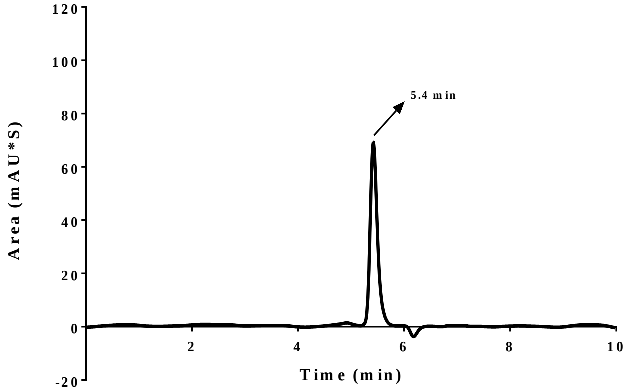

Supplementary Figure 1. GSH HPLC chromatogram.

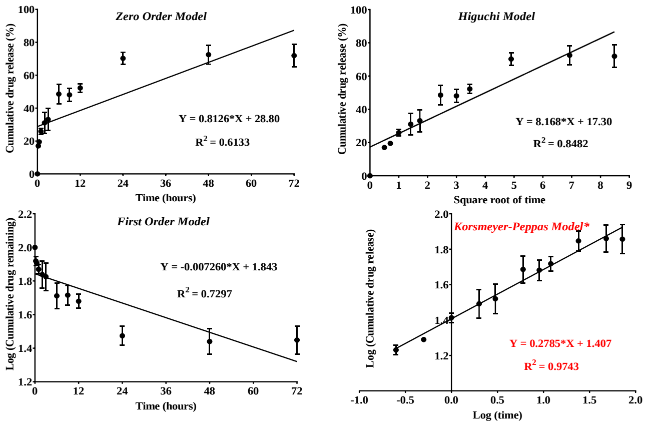

Supplementary Figure 2. GSH loaded hydrogels release profiles modelling into four release mathematical models (* the best R^2 for model selection).

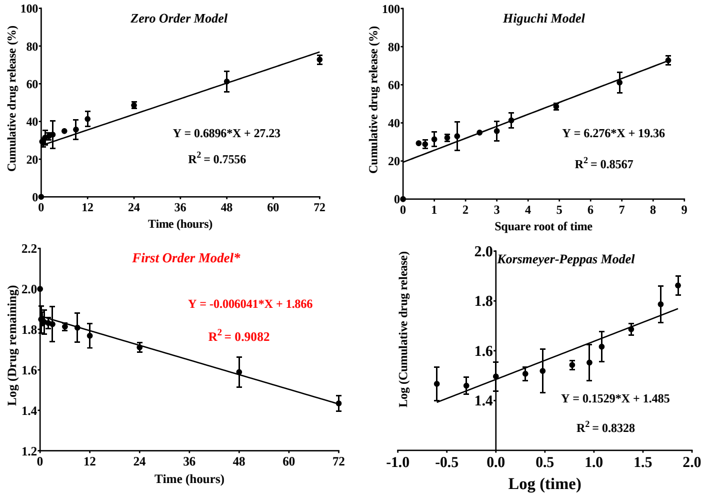

Supplementary Figure 3. GSH-SLNs suspension release profiles modelling into four release mathematical models (* the best R^2 for model selection).

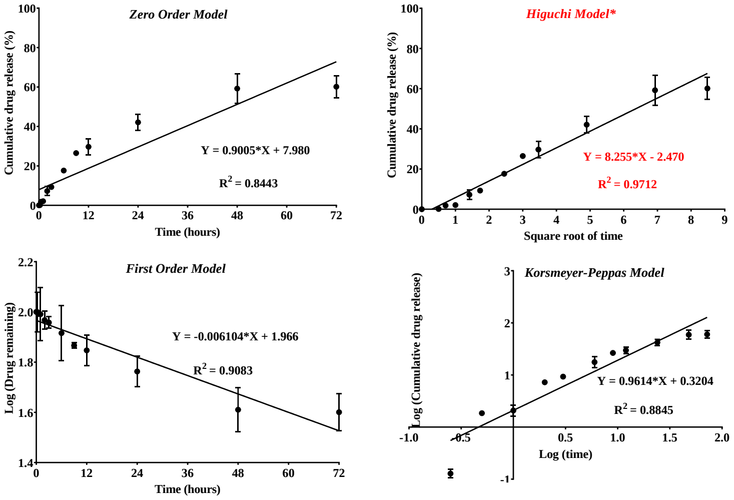

Supplementary Figure 4. GSH-SLN-EH without additional GSH in hydrogels release profiles modelling into four release mathematical models (* the best R^2 for model selection).

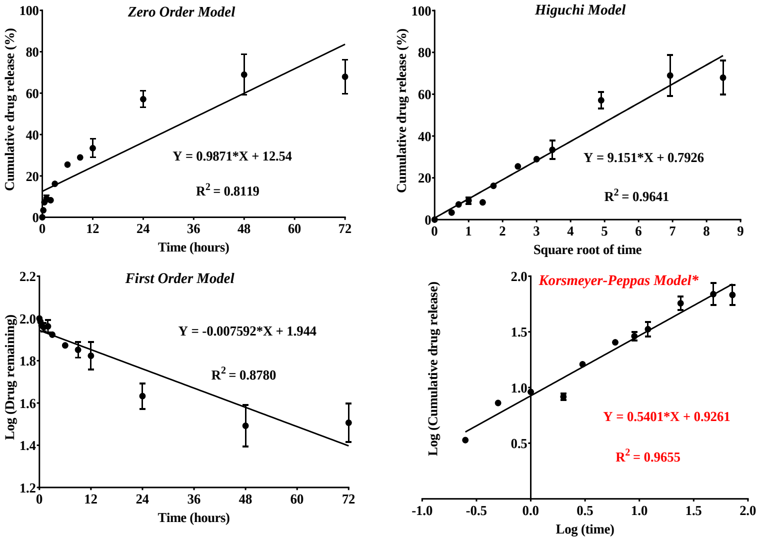

Supplementary Figure 5. GSH-SLN-EH with additional GSH in hydrogels release profiles modelling into four release mathematical models (* the best R^2 for model selection)

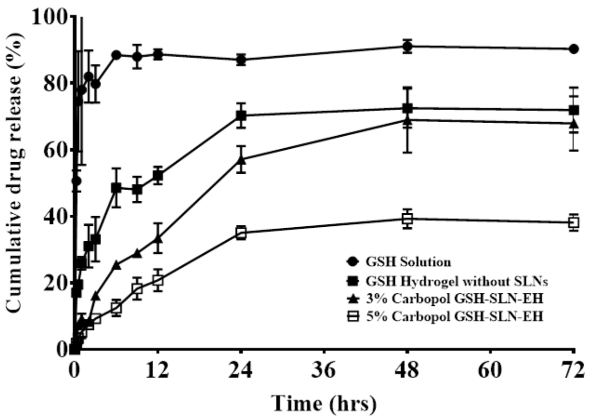

Supplementary Figure 6. Release profiles of GSH solution, GSH loaded hydrogels without SLNs, GSH-SLN-EH with 3% Carbopol and 5% Carbopol (Mean ± S.D. n = 3).

Supplementary Table 1. Gel strength, adhesion and stickiness of 3% Carbopol control gel without SLNs loaded (Mean ± S.D. n = 3).

Items	3% Carbopol control (without SLNs)
**Gel strength (g)**	4.9 ± 0.6
**Adhesion (g sec)**	2.4 ± 0.7
**Stickiness (g)**	2.1 ± 0.4
**Spreadability (g sec)**	32.9 ± 2.1

Disclaimer/Publisher's Note: The statements, opinions and data contained in all publications are solely those of the individual author(s) and contributor(s) and not of MDPI and/or the editor(s). MDPI and/or the editor(s) disclaim responsibility for any injury to people or property resulting from any ideas, methods, instructions or products referred to in the content.
